# Supplementary material for: A Differentiable Neural-Network Force Field for Ionic Liquids
Source: J Chem Inf Model. 2021 Dec 23;62(1):88–101. doi: 10.1021/acs.jcim.1c01380 (PMC8757435; doi:10.1021/acs.jcim.1c01380)
Supplement: Supplementary file 1 — ci1c01380_si_001.pdf [file ci1c01380_si_001.pdf]

Compound	SMILES	Molecular Weight (g/mol)
Ethylammonium nitrate	CC[NH3+].[N+](=O)([O-])[O-]	108.10
